# Functional recovery after surgical stabilization and postoperative radiotherapy due to metastases of long bones

**DOI:** 10.1007/s00066-018-1369-0

**Published:** 2018-09-13

**Authors:** Irenäus A. Adamietz, Michal J. Wolanczyk

**Affiliations:** 1 Department of Radiation Oncology, Marien Hospital Herne, Ruhr-University of Bochum, Hölkeskampring 40, 44625 Herne, Germany; 2 Department of Radiation Oncology, St. Josef Hospital, Ruhr-University of Bochum, Gudrunstraße 56, 44791 Bochum, Germany; 3 Department of General and Interventional Radiology and Neuroradiology, Wroclaw Medical University Hospital, ul. Borowska 213, 50-556 Wroclaw, Poland

**Keywords:** Radiotherapy, Pathological fracture, Metastatic disease, Orthopedic stabilization, Postoperative radiotherapy, Long bones, Strahlentherapie, Pathologische Fraktur, Metastasierung, Orthopädische Stabilisierung, Postoperative Strahlentherapie, Lange Knochen

## Abstract

**Purpose:**

To reinvestigate the functional recovery after combined treatment with surgery and postoperative irradiation of complete or impending pathologic fractures of long bones.

**Methods:**

We retrospectively evaluated the results of external beam radiation therapy (EBRT) carried out after 68 orthopedic stabilization procedures (femur, *n* = 55, 80.8%; humerus, *n* = 13, 19.2%) for actual or impending pathological fracture of long bone in 61 patients with skeletal metastases. The mean normalized total dose was 34.7 ± 7.8 Gy. Endpoints were patient’s functional status (FS; 1 = normal pain free status; 2 = normal use with pain; 3 = significantly limited used; 4 = nonfunctional status), a need for a secondary procedure to the same site and overall survival following surgery.

**Results:**

Overall, 75% of patients achieved normal functional status (FS 1–2) within 12 weeks after surgery. Functional recovery in surviving patients reached 93%. Median survival was 17 months (95% confidence interval 13.7–20.2). Secondary surgical intervention at the same location was necessary in 3 patients (4.4%). On multivariate analysis, only general status (*p* = 0.011) and growing potential of primary tumor (*p* = 0.049) were associated with achieving normal functional status within 12 weeks after surgery and radiotherapy. The applied radiation schemes demonstrated a comparable impact on functional recovery.

**Conclusions:**

Our results confirm the effectiveness of stabilizing surgery and fractionated postoperative radiotherapy in terms of functional recovery, supporting prior results assessing postsurgical radiotherapy versus follow-up. The patient’s general status is a strong prognostic factor for functional recovery. Rapidly growing tumors may hinder achievement of a normal functional status.

## Zusammenfassung

**Ziel:**

Untersuchung der funktionellen Wiederherstellung nach chirurgischer Stabilisierung und postoperativer Bestrahlung aufgrund von Metastasen der langen Knochen.

**Methoden:**

Wir haben bei 61 Patienten mit Skelettmetastasen und einer tatsächlichen oder drohenden pathologischen Fraktur des langen Knochens die Ergebnisse der Bestrahlung nach 68 orthopädischen Stabilisierungsverfahren (Femur, *n* = 55, 80,8%; Humerus, *n* = 13, 19,2%) retrospektiv untersucht. Die mittlere normalisierte Gesamtdosis betrug 34,7 ± 7,8 Gy. Untersuchungsendpunkte waren der Funktionsstatus des Patienten (FS; 1 = normale Funktion, schmerzfrei; 2 = normale Funktion mit Schmerz; 3 = Funktion signifikant begrenzt; 4 = nicht funktionsfähig), die Notwendigkeit der chirurgischen Reintervention an selber Stelle und das Gesamtüberleben nach chirurgischem Eingriff.

**Ergebnisse:**

Insgesamt 75% der Patienten erreichten den normalen FS (FS 1 – 2) innerhalb von 12 Wochen nach der Operation. Die funktionelle Wiederherstellung bei überlebenden Patienten erreichte 93%. Das mediane Überleben betrug 17 Monate (95%-KI: 13,7–20,2 Monate). Bei 3 Patienten (4,4%) war ein erneuter chirurgischer Eingriff an selber Stelle notwendig. In der multivariaten Analyse wurden nur der allgemeine Status (*p* = 0,011) und das Wachstumspotenzial des Primärtumors (*p* = 0,049) mit dem Erreichen eines normalen FS innerhalb von 12 Wochen nach der Operation und Strahlentherapie assoziiert. Die angewandten Bestrahlungsschemata zeigten eine vergleichbare Wirkung auf die Wiederherstellung der Funktion.

**Schlussfolgerungen:**

Unsere Ergebnisse bestätigen die Wirksamkeit der chirurgischen Stabilisierung und der fraktionierten postoperativen Strahlentherapie in Bezug auf die funktionelle Wiederherstellung und stützen frühere Ergebnisse zur postoperativen Strahlentherapie im Vergleich zu einem beobachtenden Ansatz. Der Allgemeinzustand des Patienten stellt einen starken prognostischen Faktor für die funktionelle Wiederherstellung dar. Schnell wachsende Tumoren können das Erreichen eines normalen FS behindern.

## Introduction

Orthopedic stabilization of metastatic bone lesions continues to be the mainstay for treatment of pathological bone fractures [1, 2]. The major challenge for the orthopedic procedure is to achieve stability and decrease metastatic pain in the region of the lesion or fracture. In general, pathologic fractures resulting from metastatic disease are treated by repairing or removing existing bone [3]. Intramedullary nailing or an implantation of a plate augmented with polymethylmethacrylate are the most common strategies. In the case of massive bone loss or a destroyed joint surface, the bone may be removed and replaced with a prosthesis [4, 5]. Unfortunately, tumor progression in the surgically supplied bone is common. Indeed, as stated by Townsend et al. who investigated two groups of patients with similar function before treatment, within the first 5 months following surgical intervention, only about 30% of patients treated with surgery reach normal functional status (FS) [3]. Postoperative percutaneous irradiation is necessary in most cases to eliminate residual tumor disease and thus prevent disease progression and further osteolysis [6]. Destruction of tumor cells by radiotherapy (RT) achieves pain relief, reverses inflammation resulting from bone metastasis, and promotes the ossification of lytic lesions [7]. Therefore a multidisciplinary approach and treatment including surgical stabilization, radiotherapy and systemic treatment delivers the best results [8]. The adjuvant role of postoperative RT in patients with surgically stabilized metastatic bone disease was evaluated almost 20 years ago by only one study performed by Townsend et al. The observed proportion of patients reaching normal FS (± pain) at any time was 53% for the group having surgery plus postoperative RT versus 11.5% for the surgery-only group. The later published studies examined the number of surgical reinterventions and complications [9, 10]. The functional outcome has not been investigated since then.

As treatment strategies and supportive care have improved throughout the years, we re-evaluated the results of surgery and postoperative external beam radiation therapy (EBRT) in patients with surgically stabilized metastatic bone lesions.

## Patients and methods

Between January 2003 and February 2012 a total of 1101 patients with metastatic bone disease (identified in the hospital database as having ICD code C79.5, regardless of the primary site) were treated with EBRT. Of this group, 126 patients (11.4%) had undergone a prior orthopedic stabilizing procedure for impending or actual fracture. The medical record, obtained from clinical charts of different departments involved in treatment (i.e., from admissions, surgical, internal, and radiation therapy departments and also from lab and imaging studies), was reviewed and used as a basis for evaluation of attainment of normal FS, a need for a secondary orthopedic procedure and for overall survival following surgery.

The following inclusion criteria were established for the selection of eligible patients: the presence of an actual or impending pathological fracture of a long bone caused by bone metastasis treated with surgery and followed by postoperative EBRT; bone metastasis proven by biopsy; the presence of a metal stabilizing device with or without the use of bone cement implanted on surgery; and no previous RT to the fractured site. The medical history had to contain detailed information on complete personal data, complete data on the patient’s functional and general status with a fully described orthopedic examination, outcomes of postoperative RT, and medication used. Exclusion criteria were: patient’s very poor general status after surgery, an incomplete medical record, especially regarding information on the follow-up examinations and a lack of reason for referral for surgery or RT.

According to the inclusion/exclusion criteria, 1040 medical histories were rejected. Finally a total of 61 patients who underwent an orthopedic stabilizing procedure for a metastatic long bone lesion prior to irradiation were evaluated. The functional outcome of 68 surgical interventions in femur (*n* = 55, 80.8%) and in humerus (*n* = 13, 19.2%) was analyzed (7 patients were operated on at two sites). Table 1 shows baseline characteristics for all included patients.

**Table 1:** Characteristics of investigated group and classification of potential prognostic factors for functional recovery

Related factor	Subcategory	No. of sites/%
*Gender*	Male	31/45.5
	Female	37/54.5
*Age (years)*	≤70	36/52.9
	>70	32/47.1
*Karnofsky performance status*	>60	40/58.8
	≤60	28/41.2
*General status at the beginning of radiation therapy*	1 group: good or slightly reduced	33/48.5
	2 group: any worse than in group 1	35/51.5
*Primary tumor lesion from which cells have metastasized to bone*	1 rapid growth group (expected survival <10 months)	25/36.7
	2 slow growth group (expected survival >10 months)	43/63.2
*Visceral or cerebral metastases*	Yes	30/44.4
	No	38/55.8
*Previous chemotherapy*	Yes	45/66.1
	No	23/33.9
*Location of skeletal metastases*	Femur	55/80.8
	Humerus	13/18.2
*Method of surgical stabilization*	Plate and screws	30/44.1
	Intramedullary nailing	38/55.8
*Complete pathological fracture*	Yes	48/70.5
	No	20/29.5
*Resection of the secondary site*	No resection	34/50.0
	Complete or partially complete	29/42.6
	Unknown	5/7.4
*Osteolytic character*	Yes	48/70.5
	No	20/29.5
*Bisphosphonates*	Yes	39/57.3
	No	29/42.7
*Inclusion of the entire stabilizing device within the range of target volume*	Yes	36/52.9
	No	32/46.1
*Number of target volumes irradiated in postsurgical RT * *(in case of multiple also extraosseous sites were treated)*	One	37/54.4
	Multiple	31/45.6
*Received normalized total dose*	TD >35 Gy	33/48.5
	TD ≤35 Gy	35/51.5

*RT* radiotherapy, *TD* total normalized dose

### Functional status

Patients in our study were categorized into one of four groups, depending on their FS according to the assessment method described previously by Townsend et al. in 1995 [3]: FS 1 = normal, pain-free status; 2 = normal or near normal use with pain; 3 = significantly limited use or movement, alternatively requiring some type of prosthesis (e.g., crutches, walker rollator); and 4 = nonfunctional status (e.g., bedridden, wheelchair bound). To assess whether patients could walk or use the affected extremity in a meaningful way following surgery and irradiation, we combined patients with FS 1 and 2 (normal function), describing normal FS (± pain), and compared them to the grouped patients with FS 3 and 4 (reduced function). Data on patient FS was gained retrospectively covering four different periods: the outset of RT, which fell close (up to 3 days) to application of the first radiation fraction, and during 1–3, 3–6, 6–12, and 12–15 weeks of the postoperative period. The effectiveness of surgery and EBRT was measured in terms of the patient fraction that reached normal function (FS 1 or 2) within 12 weeks (3 months) following the surgical intervention.

### Treatment

Postoperative EBRT was defined as radiation treatment that started within 8 weeks postsurgery (mean: 4.83 weeks). A decision to refer the patient both for surgery and postoperative RT was made principally by an interdisciplinary board after the patient had been sent for a consultation either by the treating orthopedic surgeon or oncologist. This decision was always confirmed by detailed and comprehensive description of patient’s general and local status within clinical charts. Surgical stabilization of long bones was performed either with bone plate and screws (30/44.1%) or intramedullary nailing (38/55.8%). All patients were treated with a linear accelerator using a 6 MV or 15 MV photon beam. Target volumes were determined using different techniques including the following setups: single beam (15/22.0%), two beams (35/51.4%), and 3D-conformal beams (18/26.6%). The total dose (TD) ranged from 6 to 56 Gy (mean dose, 31.2 Gy ± 10.5). The prescribed fractionation schemes were 10 × 3 Gy (*n* = 26), 20 × 2 Gy (*n* = 20), and 14 × 2.5 Gy (*n* = 21). Two patients terminated radiotherapy due to medical nontumor-associated reasons before the planned total dose was achieved (one at 6 Gy and one at 14 Gy). One patient with lung cancer received 56 Gy (28 × 2 Gy) because of tumor bulk around the fracture. The normalized TD (single dose of 2 Gy) was calculated using the alpha/beta model assuming an alpha/beta value of 10. The mean normalized TD was 34.7 ± 7.8 Gy (95% confidence interval [CI]: 33.0–36.5 Gy).

### Follow-up

All patients were followed until death. The mean follow-up period was 16.3 months (range: 2 weeks–29 months).

### Prognostic factors

Factors examined for their prognostic significance, shown in Table 1, were evaluated according to a yes/no scale to allow an easier evaluation of their possible influence on patient functional independence (reaching FS 1 or 2). Most factors were evaluated as they were observed close to application of the first radiation fraction. All primary sites were classified into two groups derived from the classification described by Katagiri et al. [11]. All primary malignant entities with expected survival lower than 10 months were classified as rapid growing, the remaining primaries as slowly growing. For the analysis of total radiation dose patients missing the prescribed dose were excluded.

### Statistics

All calculations were done with IBM SPSS 25 (SPSS Inc., Chicago, IL, USA) package software. In addition, two basic distributions and calculations of standard statistical parameters were performed. The differences between frequencies were analyzed using the chi-square test, and prognostic factors were calculated according to the Cox regression model. For the regression analysis, only variables with a distribution of at least 25 cases per group were included. The selection of variables for multivariate analysis required a *p* ≤ 0.1 on the univariate analysis. Time table analyses of FS and survival were performed using the Kaplan–Meier method. Statistical differences were calculated by the log-rank method. *P*-values <0.05 were classified as significant; values <0.01 as highly significant; and values between 0.05 and 0.15 were interpreted as indicating a statistical trend.

## Results

### Assessment of FS

**Fig. 1:**
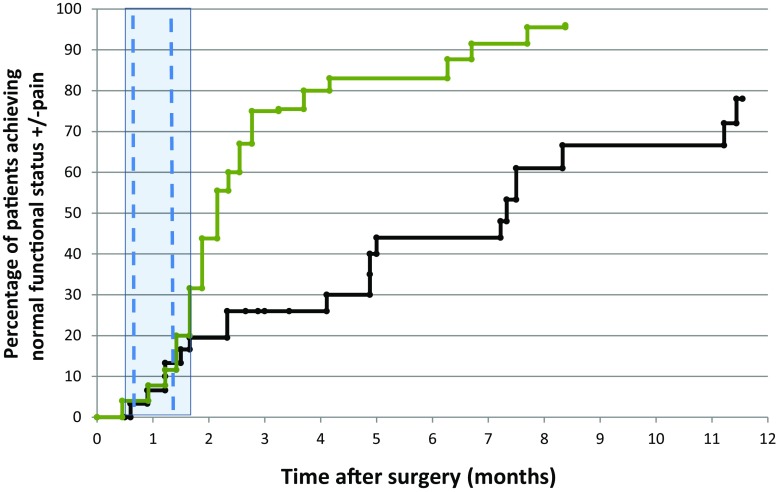
Normal functional status (± pain) following surgery and irradiation in the investigated group (*green line*) against results observed in the historical reference group (*black line*) (surgery + RT [radiotherapy], redrawn from [3]). The *vertical blue lines* mark the onset and the end of radiation treatment in 75% of patients; the *blue zone* covers the treatment duration of all sites

The evaluation of patients reaching FS 1 or 2 (normal FS [± pain]) was conducted for 68 sites in the study group. The observed proportion of patients reaching FS 1 or 2 within the first 12 weeks following surgery was 76%. Recovery to normal function (± pain) at any time was observed in 45 patients (66.1%). The mean time needed for functional recovery was 2.74 months (95% CI: 2.17–3.31 months). The median was 2.07 months (95% CI: 1.77–2.36 months). The recovery started a few weeks after the surgical intervention and seemed to continue without interruption during irradiation (Fig. 1).

Functional response to RT was also assessed in regard to proportion of patients reaching normal FS (± pain) under different radiation regimens. The applied regimes demonstrated a comparable impact on functional recovery. There was no correlation between total dose and functional outcome.

At the univariate level, primary lesion group (*p* = 0.008), general status (*p* = 0.01), and applied normalized TD >35 Gy vs. ≤35 Gy (*p* = 0.034) were potential predictors of patients attaining FS 1 or 2 after surgery. Karnofsky score >60% vs <60% (*p* = 0.056), previous chemotherapy (*p* = 0.133), bisphosphonates (*p* = 0.141), number of target volumes in postsurgical RT (one vs. multiple; *p* = 0.146), gender (*p* = 0.147), and visceral or cerebral metastases (*p* = 0.147) showed a statistical trend. Nonsignificant findings were associated with age, location of skeletal metastasis, complete pathological fracture, method of surgical stabilization, resection of the secondary site, the osteolytic character of the lesion, and inclusion of the entire stabilizing device within the range of target volume.

On multivariate analysis, only general status at the beginning of RT (*p* = 0.011) and primary lesion group (*p* = 0,049) were associated with achieving normal function following surgery and radiotherapy.

### Second orthopedic procedure

For 68 investigated sites, second stabilizing procedures were required at the same site in 3 (4.4%) of patients. Although the reason for these procedures was not clearly stated in the available medical record, in 2 (2.9%) patients, the loosening appeared to be caused by a further development of metastatic bone lesions in the operated region. A total of 6 (8.8%) patients required a further orthopedic procedure at different locations than the site in question.

### Overall survival of the investigated group

**Fig. 2:**
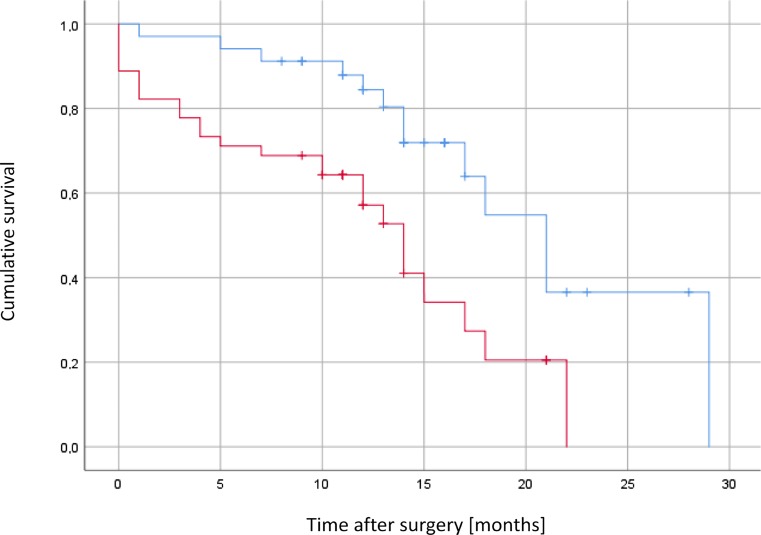
Overall survival in the surgically stabilized group followed by radiation therapy (*n* = 61). Patients (*n* = 26) in good or slightly reduced general condition (*blue line*), patients (*n* = 35) any worse than in the other group (*red line*)

Twelve months after surgery, 67% of patients were still alive. The mean survival in the overall group was 16.3 months (95% CI: 13.8–17.9, median: 17.0 months; 95% CI: 13.7–20.2). The general status of the patient had an impact on survival (Fig. 2).

## Discussion

Metastatic bone disease leads to deterioration of bone metabolism with a subsequent loss of bone mechanical function combined with severe pain. An actual fracture or a threatening metastatic bone lesion, therefore, makes a prophylactic or interventional stabilization by surgery essential in the management of skeletal metastases. A residual tumor mass remaining in the bone marrow and in the vicinity of stabilizing material provides excellent conditions for malignant re-growth after surgery, resulting in a relapse and deterioration of function. Adjuvant irradiation after surgical intervention in bone metastases can effectively eliminate residual tumor cells allowing for local pain reduction and long-lasting recalcification of bone [12–14].

Townsend et al. [3] reviewed 64 orthopedic stabilization procedures in 60 consecutive patients with metastatic disease and previously unirradiated weight-bearing bones (91% femur) with pathological or impending pathological fracture. A total of 35 sites that received adjuvant RT were compared to 29 sites that were treated with surgery alone. Authors stated that postoperative RT is the most important factor in patients achieving and maintaining a normal functional status. In that study, postoperative RT was also associated with fewer orthopedic procedures as well as with an improved overall survival.

Two years later Van Geffen et al. [9] published a retrospective analysis on survival and functional results after operative therapy of pathological fractures. A total of 110 fractures were treated operatively (72%), 27 with irradiation (18%), and 15 were treated conservatively (10%). Of all operated patients, postoperatively 79% regained walking capacity and 60% required no or only occasionally analgesic drugs. Bone-related complications included failure of the osteosynthetic device or prosthetic implant as well as progression or re-occurrence of the disease within the operative field. In the non-irradiated group, 21% had complications vs. 14% in the patients who had received additional radiotherapy. The difference was remarkable but not statistically significant.

More recently, Drost et al. [10] published a retrospective analysis on need for second surgery, rates of re-irradiation, tumor progression and prosthesis displacement following postoperative radiation of infiltrated bone. Data were collected from 65 patients who received postoperative radiation to 74 sites in the extremities. Only 2 patients required a second surgery (2.7%) at 9 and 10 months after postoperative radiation. Pain increase requiring re-irradiation was reported in 7 patients (9.5%), at a median time of 9.3 months after the delivery of radiation. Of the 47 patients who had radiological imaging available, local progression of bone metastases was seen in 8 patients (17.0%) and displacement of the prosthesis in 1 patient (2.1%).

The current data confirm the efficiency of surgery combined with postsurgical fractionated external beam irradiation. In the presently investigated group the number of patients regaining normal function following surgery was higher and the functional recovery was achieved faster than in the publication by Townsend et al. [3]. The results of postoperative RT in our patient population suggest that about 80% of patients may achieve a good functional result within 3–4 months after surgery (Fig. 1). Similarly elevated functional recovery rates after surgical stabilization were reported by van Geffen et al. [9]. The difference in outcomes seems to depend on patient selection for adjuvant RT.

Other explanations for the observed differences are more speculative, but seem to be quite reasonable, and the improved operative procedure and supportive treatment must be taken into account. Especially minimally invasive surgical techniques, early postoperative verticalization of patients, more accurate imaging techniques allowing for more precise assessment and adjustment of postoperative therapy should be considered.

Although a stabilizing and protective effect is achieved immediately after surgery, it is because of the altered metabolism of bone that successful remodeling following radiation treatment requires several weeks to be effective. This period seems to be reflected by the flatter part of the graph, observed within first 3–4 weeks following the onset of RT. According to our results (Fig. 1) the time needed for appropriate bone remodeling following RT did not change and still equals a few weeks (overlapping graphs seen during the first 3 weeks following the onset of RT). The assumption that the optimized radiation techniques are responsible for the better result is less likely and needs to be considered with great caution. Possible bias in reference of current results to Townsend’s study may result from slightly different sites of treatment in both groups (91% of femur in [3] vs. 80.2% in the current study).

Because of the high uptake of the method and wide range of literature supporting the benefit of metastatic bone disease irradiation [4, 6, 7, 10, 14, 15], currently almost all patients are irradiated postoperatively. Willeumier et al. [16] published a systematic review of literature on postoperative irradiation after surgical bone stabilization. The review was based on two articles [3, 9] and identified 64 and 110 patients of whom 55% and 28% received postoperative RT, respectively. They concluded that the current available literature might be insufficient to decide whether postoperative RT after surgical stabilization should be standard care.

Due to high grade of acceptance of adjuvant irradiation, currently only patients in a very poor general status at fracture presentation or after surgery do not receive postsurgical RT as not to cause further weakening of their condition. This makes it very difficult nowadays to collect an appropriate comparator group of patients who would be subjected to surgical stabilization only (without adjuvant RT) being postoperatively in similar general condition (i.e. not significantly worse) as the irradiated group. Thus we believe no reliable comparative assessment can be done nowadays to quantify the exact contribution of the postoperative RT.

Our results do not support an impact of total dose on functional recovery. The tolerance dose of the bone is given as 40 Gy in 4 weeks [17]. Exceeding that dose level initiates trophic changes of bone that may inhibit skeletal recovery. Since recalcification of bone is the aim, fractionated irradiation is advocated [18]. Therefore, a dose range between 35 Gy and 40 Gy seems to be appropriate to generate good treatment results.

We conclude that fractionated radiotherapy following surgical stabilization of bone impaired by metastatic disease is effective in terms of functional recovery. Within 3–4 months almost 80% of patients functionally recover. The probability of recovery can be limited by reduced general condition of the patient. Rapidly growing tumors might also hinder achieving a normal functional status. Hence, postoperative radiotherapy after surgical bone stabilization should be critically discussed in generally reduced patients, especially when bearing progressive tumors.
